# Effect of Seed Priming with Endophytic *Bacillus subtilis* on Some Physio-Biochemical Parameters of Two Wheat Varieties Exposed to Drought after Selective Herbicide Application

**DOI:** 10.3390/plants12081724

**Published:** 2023-04-20

**Authors:** Oksana Lastochkina, Albina Yakupova, Irina Avtushenko, Artem Lastochkin, Ruslan Yuldashev

**Affiliations:** 1 Institute of Biochemistry and Genetics UFRC RAS, 71 Pr. Oktyabrya, 450054 Ufa, Russia; 2 Department of Biology, Ufa University of Sciences and Technology, 32 Zaki Validi, 450076 Ufa, Russia

**Keywords:** *Triticum aestivum* L., stress combination, herbicide, drought, tolerance, endophytic *Bacillus subtilis*, plant–microbe interaction

## Abstract

Wheat plants are frequently exposed to combined herbicide and drought stress (HDS) which induces complex responses negatively, affects productivity, and is becoming more exacerbated with current climate change. In this work, we studied the influence of seed priming with endophytic bacteria *Bacillus subtilis* (strains 104 and 26D) on growth and tolerance of two wheat (*Triticum aestivum* L.) varieties (E70—drought tolerant; SY—drought susceptible) exposed to soil drought after application of selective herbicide Sekator^®^ Turbo in pot experiments under controlled conditions; 17-day-old plants sprayed with herbicide and after 3 days were subjected to soil drought by stopping irrigating the plants for 7 days with subsequent resumption of normal irrigation (recovery). Additionally, the growth of tested strains (104, 26D) in the presence of different concentrations of herbicide Sekator^®^ Turbo and drought (PEG-6000) were evaluated. It was established that both strains are herbicide and drought tolerant and capable to improve seed germination and early seedlings’ growth under different herbicide and drought stress degrees. The results of pot experiments showed that HDS exposure declined growth (plant length, biomass), photosynthetic pigments (chlorophyll a and b), leaf area, and increased lipid peroxidation (LPO) and proline accumulation in plants, demonstrating higher damaging effects for SY variety. Strains 104 and 26D mitigated (in different levels) such negative impacts of HDS on growth of both varieties by increasing length of roots and shoots, biomass, photosynthetic pigments (chlorophyll a and b), and leaf area, reducing stress-caused LPO (i.e., malondialdehyde), and regulating proline biosynthesis, as well as contributing to a faster recovery of growth, photosynthetic pigments, and redox-status of plants in post-stress period in comparison with non-primed plants. These ultimately manifested in forming a better grain yield of both varieties primed with 104, 26D, and exposed to HDS. Thus, both strains 104 and 26D (which are herbicide and drought tolerant) may be used as seed priming agents to improve wheat HDS tolerance and grain yield; however, strain 104 more effectively protected plants of E70, while strain 26D—plants of SY. Further research should be focused on understanding the mechanisms that determine the strain and variety-specificity of endophytic symbiosis and the role of bacteria in the modulation of physiological states of primed plants under stress conditions, including HDS.

## 1. Introduction

Soft spring wheat (*Triticum aestivum* L.) is the most important cereal and bread crop, which is of great importance in ensuring the food security worldwide [1]. Losses of grain yield/quality from major abiotic stresses such as drought and its combinations with other stresses, cause a significant damage to agriculture, food industry, and economy. Drought, adversely affecting the overall plant metabolism at the physiological, biochemical, and molecular levels, leads to the damages of various cellular compartments, protein degradation, enzyme inactivation, reduced nutrient uptake, transpiration and photosynthesis rates, stomatal closure, growth inhibition, and wilting and drying of plants [2,3,4,5].

In modern wheat cultivation technologies, the use of herbicides for weed control plays an important role in practical (commercial) agriculture, but creates additional pressure on plants and, in combination with drought, has a detrimental effect on crop yields, which is becoming increasingly tangible under the changing climate [6,7,8]. Herbicides are a major environmental problem, mainly due to their ability to penetrate the metabolic pathways of plants and microorganisms [9]. Rapidly absorbed by the leaf surface and plant root system, herbicides are able to move freely along with nutrients and accumulate in growth points, affect photosynthesis or photosynthetic pigments, biosynthesis of auxin, amino acids, and fatty acids. Under the action of herbicides, an increase in the energy respiration, a decrease in the intensity of photosynthesis and enzyme activity, inhibition of chlorophyll synthesis, a change in the ratio of forms of soluble sugars and N compounds, and inhibition of protein synthesis in plants are usually noted [10]. In fields of spring wheat crops, they often use highly selective herbicides against annual and some perennial bipartite weeds. These, for example, include the herbicide Sekator^®^ Turbo (active ingredients: iodosulfuron methyl sodium, amidosulfuron, and mefenpyrdiethyl (antidote)) (Bayer AG, Frankfurt, Germany). The innovative formulation provides greater reliability against hard-to-eradicate weeds, overgrown weeds, and weeds treated in difficult weather conditions. The use of herbicides is generally not recommended for crops under such abiotic stresses as drought and is often explicitly stated in the manufacturer’s instructions for use. However, drought is a recurring phenomenon in almost all wheat growing regions where herbicide application is a common practice [11]. An increase in the number of factors simultaneously affecting plants, their survival and growth is reduced, even if the levels of each of these individual stressors are low [7]. The combined effect of herbicides and drought (HDS) caused significant morphophysiological and biochemical features of plants including growth suppression, reduced photosynthesis and leaf area, altered oxidative metabolism, membrane instability, stomatal conductance, increased oxidative stress markers, altered root growth, and disturbed water relations resulting in diminished growth and yield [8,11,12,13]. Thus, drought in combination with herbicide application has a synergistic negative influence on plant growth and yield [14], and is becoming more exacerbated with current climate change.

Beneficial plant growth-promoting microorganisms (PGPMs) capable of activating the natural protective mechanisms of host plants are regarded as an affordable and eco-friendly strategy to increase adaptive capacity and preserve crop yield in a stressful environment [15,16,17,18,19]. Among PGPMs, a special interest are *B. subtilis* which are widely recognized as safe microorganisms for use in the food industry [15,20,21]. Moreover, *B. subtilis* producing endospores are extremely tolerant to different physical and chemical effects (heating, drying, organic solvents, UV-irradiation, etc.), making them attractive for the development of commercial biologicals for plant protection. Moreover, endophytic *B. subtilis*, living within plant tissues, may be the most effective in protection of plants against adverse environmental factors allowing them to be less dependent on external environmental factors (compared to rhizospheric and phyllospheric strains) simultaneously exhibiting beneficial properties [22,23,24], being ideal agents to develop microbial-based biologicals. To date, a large body of data has been gathered on the ability of PGPMs, including endophytic *B. subtilis*, to directly/indirectly improve the growth and development of different plant species at a normal rate and at the impact of various biotic and abiotic stress factors, thereby increasing yields and product quality [21,25]. The positive influence of PGPMs to cope with drought in wheat plants are well documented and recently reviewed in detail [15,25]. Physiological and anti-stress action of PGPMs on plants is associated with improved mineral nutrition [26], water metabolism, photosynthesis [27], products of a set of biologically active antibiotic compounds [28,29], phytohormones [30], osmoregulator and antioxidant [31,32,33] activities, modulation of endogenous phytohormones [34,35], and induction of plants system resistance/tolerance [27,28,33]. However, in practice, their effectiveness often varies depending on many factors (characteristics of the strain, type of plant, its ecological and geographical origin and place of growth, varietal characteristics, types of stress that plants are exposed to during the growing season, etc.) [32,36,37]. So, currently, one of the main constraints to the development and implementation of endophyte-based biologicals is the lack of knowledge about the mechanisms underlying their interactions with plants under stress and/or their combinations. It is not yet clear how endophytic *B. subtilis* regulate the protective system of host plants under combined HDS. Since the opening and installation of the role of PGPMs in the regulation of anti-stress physiological programs in plants, special attention was paid to the reaction of plants to the treatment with PGPMs under the influence of individual stress and little attention was paid to the issue of combined effects of various stresses. Only recently, the use of PGPM strains to protect plants against herbicide toxicity [38,39,40,41,42] alone and in combination with other stresses has been considered in several publications [43,44,45,46,47]. The studies devoted to the effects of PGPMs on wheat under combined HDS are also at the initial stage. Only recently was it reported that application of PGPMs are capable of mitigating HDS-caused damages on wheat plants [38,47]. There is no information how endophytic *B. subtilis* interact with wheat plants contrasting in susceptibility to dehydration under combined HDS, which determines the relevance of researches in this direction.

The aim of this study was to evaluate the effect of seed priming with endophytic bacteria *B. subtilis* (strains 104 and 26D) on growth and tolerance (some physio-biochemical parameters) of two wheat varieties, differing in drought susceptibility, under combined HDS and post-stress period (recovery).

## 2. Results

### 2.1. Herbicide and Drought Tolerance Test

**Figure 1:**
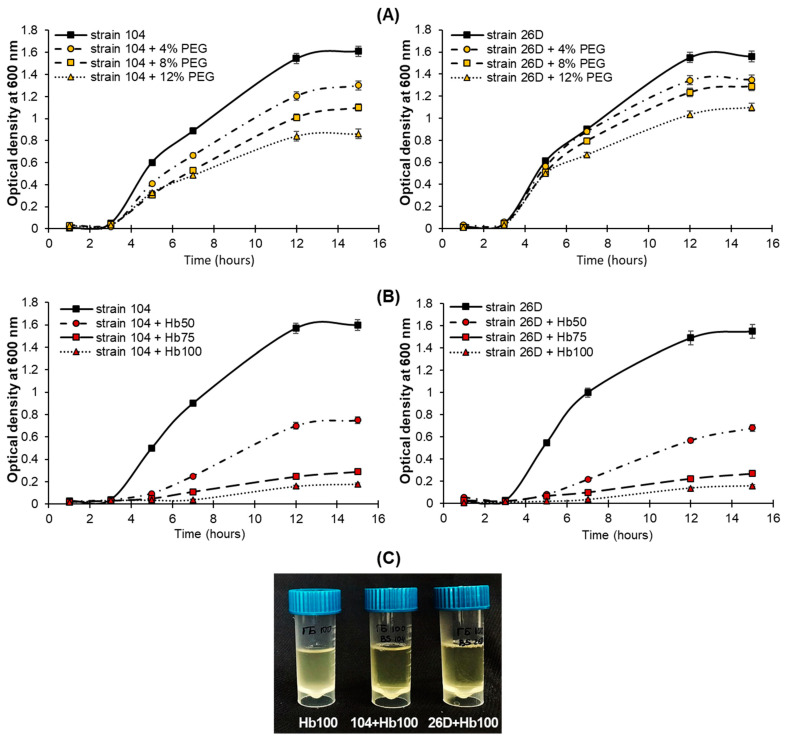
Growth curves of *Bacillus subtilis* strains 104 and 26D in control liquid Luria-Bertani (LB) medium and with the addition of herbicide Sekator^®^ Turbo (0.25 mL L^−1^ (Hb50), 0.375 mL L^−1^ (Hb75), and 0.5 mL L^−1^ (Hb100)) (**A**) and PEG-6000 (4%, 8%, and 12%) (**B**); Photographs representing the ability of bacteria to clear the solution of LB medium containing 0.5 mL L^−1^ herbicide (Hb100) after incubation for 24 h (**C**). The bacteria were incubated at 37 °C (180 rpm) for 24 h. The bacterial growth was monitored by measuring the optical density at 600 nm. Data were obtained in triplicate for each group. Error bars represent standard errors (±SE) of the means.

The evaluation of tolerance to different concentrations of Sekator^®^ Turbo herbicide (Bayer AG, Frankfurt, Germany) and drought (PEG-6000) showed that the tested strains *B. subtilis* 104 and 26D were both herbicide and drought tolerant. It was revealed that there was a concentration-dependent decline in bacterial cells’ growth over the cultivation period (Figure 1A,B), but the strains 104 and 26D remained at capacity to grow (although slower than the controls) both in the presence of PEG (Figure 1A) and herbicide (Figure 1B) on liquid Luria-Bertani (LB) growth medium. The results based on OD data also showed that both strains were more tolerant to drought (Figure 1A) than to herbicide (Figure 1B). Additionally, it was observed that strains 104 and 26D were capable to clear the solutions of LB medium containing herbicide (Figure 1C), probably due to degrading pesticides in growth media over the incubation. It was noticed that only after clearing the growth medium that the strains began to grow (Figure 1B). This process was dose-dependent, i.e., with increasing the dose of herbicide, the strains took more time to clear herbicide and start growth. Thus, the results indicate that tested strains 104 and 26D along with tolerance to herbicide are also able to accelerate herbicide degradation.

### 2.2. Effect of Herbicide Sekator^®^ Turbo-Stress and PEG-Stress on Seed Germination and Early Hydroponic Seedling Growth

**Figure 2:**
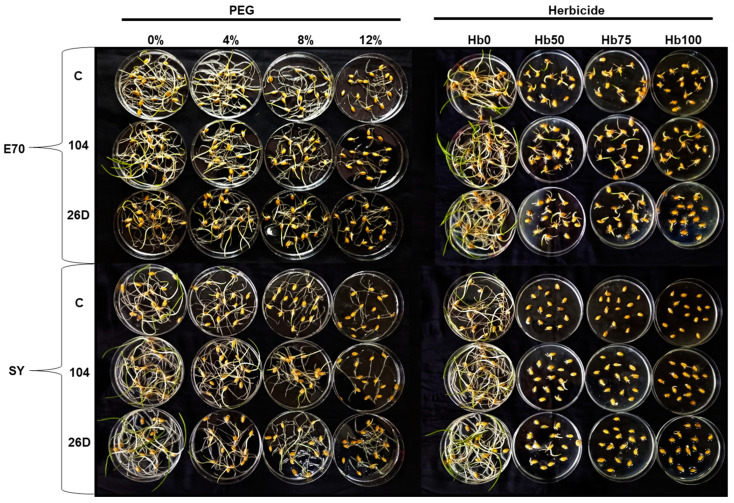
Effects of different concentration of herbicide Sekator^®^ Turbo (0 mL L^−1^ (Hb0), 0.25 mL L^−1^ (Hb50), 0.375 mL L^−1^ (Hb75), 0.5 mL L^−1^ (Hb100)), and drought (PEG) (0%, 4%, 8%, 12%) stresses on bacterial (strains 104 and 26D) inoculated seed germination in wheat after 7 days of treatments.

Seed germination and early hydroponic seedling growth of wheat were significantly affected by various concentrations and durations of herbicide Sekator^®^ Turbo (0 mL L^−1^ (Hb0), 25 mL L^−1^ (Hb50), 0.375 mL L^−1^ (Hb75), and 0.5 mL L^−1^ (Hb100)), and by drought stress induced by various concentrations of PEG-6000 (0%, 4%, 8%, and 12%) (Table 1, Figure 2). It was revealed that responses of seeds were varied based on concentrations and durations of stressors. With increase in the concentration of stressors’ seed germination and early seedlings’ growth, root and shoot length gradually decreased (by times) in both wheat varieties. With that, the seeds of E70 had better germination percentage and growth than SY on high PEG and especially herbicide gradients. Strains 104 and 26D ameliorated the negative impact of drought- and herbicide-caused stresses on seed germination in comparison with control uninoculated groups. In a similar manner, strains 104 and 26D improved (up to 20–30%) early seedling growth under herbicide and drought stresses showing the best results for E70. Thus, E70 variety showed better tolerance both to herbicide and drought stresses, and strain 104 showed better protection of E70 and SY against herbicide stress.

Representative photographs of wheat germination post priming with bacterial strains under different PEG and herbicide (Hb) concentrations are presented in Figure 2.

### 2.3. Growth of Wheat Plants Sprayed with Herbicide Sekator^®^ Turbo and Exposed to Soil Drought in Pot Experiments

**Figure 3:**
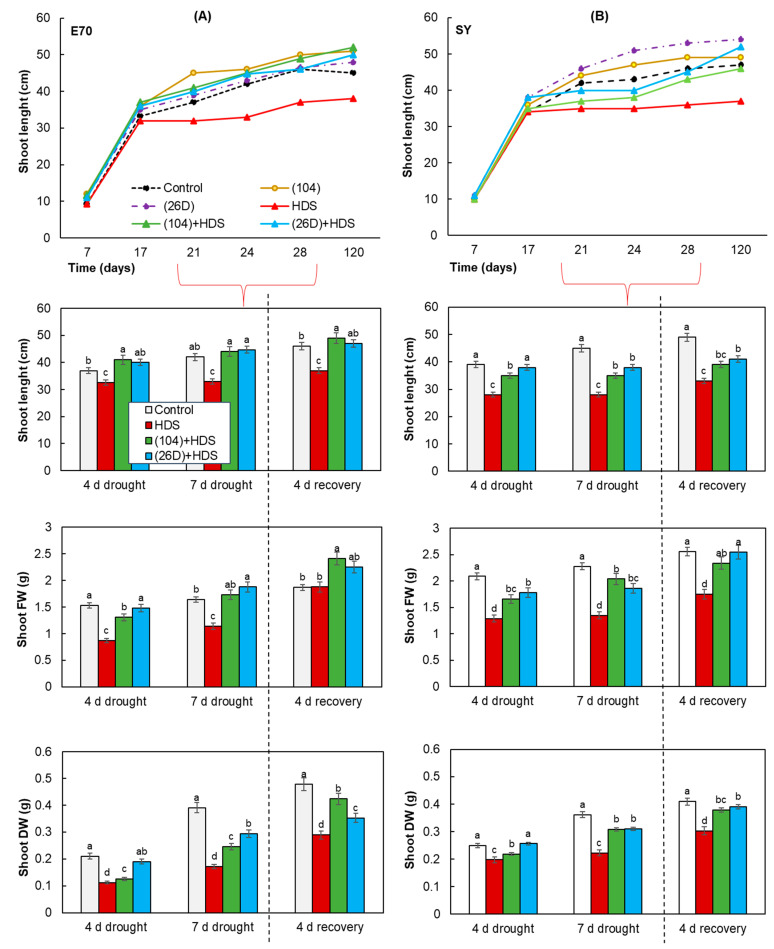
Influence of *B. subtilis* 104 and 26D on growth (length, fresh, and dry weights) of underground part (shoots) of two spring wheat varieties E70 (**A**) and SY (**B**) under combined herbicide + drought stress (HDS), as well as in the post-stress period (4 d recovery) after the resumption of normal watering. Error bars represent standard errors (±SE) of the means in triplicate. Growth parameters were measured using 25 plants from each replicate. Various letters show a significant difference between the averages of different groups at *p* < 0.05.

**Figure 4:**
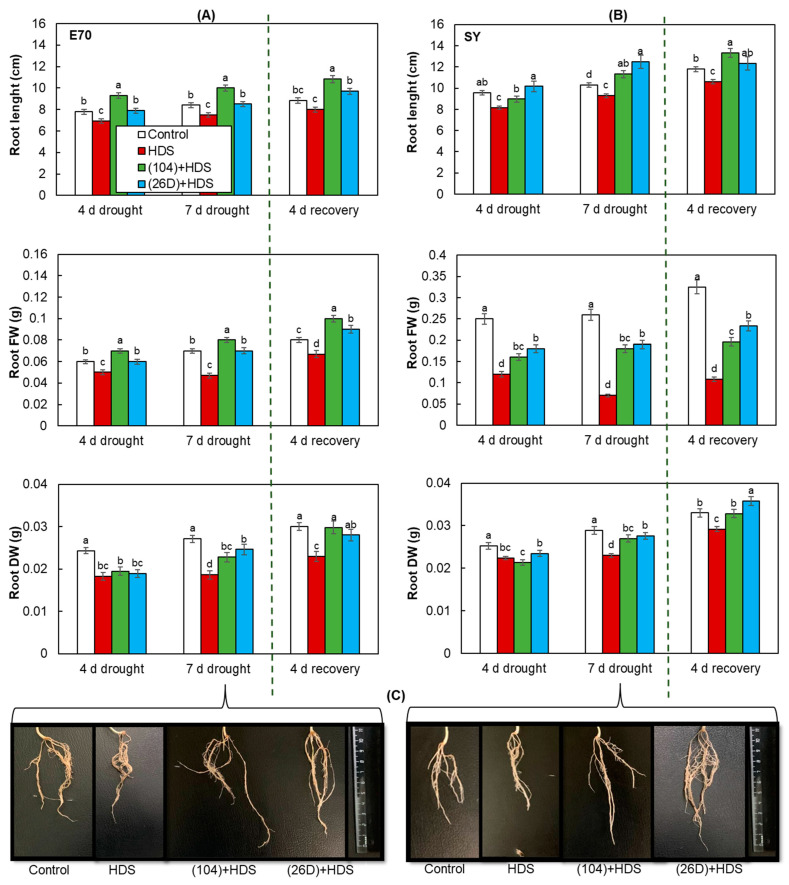
Influence of *B. subtilis* 104 and 26D on root growth (length, fresh, and dry weights) of spring wheat varieties E70 (**A**) and SY (**B**) under combined herbicide + drought stress (HDS) and the post-stress period (4 d recovery) after the resumption of normal watering; (**C**) visual appearance of wheat roots inoculated with bacterial strains (104, 26D) and after herbicide was sprayed and exposed to drought for 7 days. Error bars in figures represent standard errors (±SE) of the means in triplicate. Growth parameters were measured using 25 plants from each replicate. Various letters show a significant difference between the averages of different groups at *p* < 0.05. HDS—herbicide + drought stress.

**Figure 5:**
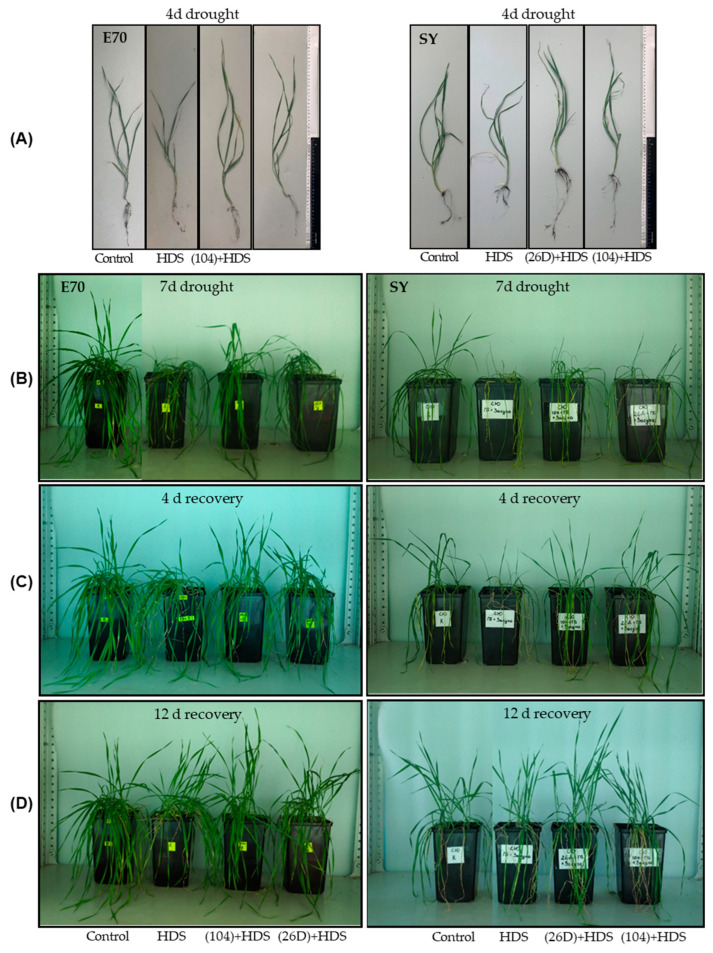
Influence of *B. subtilis* 104 and 26D on the phenotypic characteristics of wheat plants of varieties E70 and SY sprayed with herbicide Sekator^®^ Turbo and exposed to soil drought for 4 and 7 days with the subsequent resumption of normal irrigation. (**A**) 4 days of drought exposure; (**B**) 7 days of drought exposure; (**C**) 4 days of recovery after resumption of normal irrigation, (**D**) 12 days of recovery.

The effects of a combination of herbicide + drought stress (HDS) have inhibited the growth rate of shoots, roots of both varieties, and led to the reduction of their FW and DW (Figure 3, Figure 4 and Figure 5). Significant wilting and growth slowdown (by 25–40%), and reduction of FW (by 30–60%) and DW (by 30–50%) were observed in herbicide-sprayed plants of E70 and SY varieties after 7 days of soil drought exposure. It is also observed that an amplitude of stress-caused decline in the length of shoots (by 40%), shoots FW (by 41%), and roots FW (by 60%) was more pronounced in SY variety than in E70. These demonstrate that SY variety is more sensitive to HDS than E70. Additionally, it was discovered that after such a short time (7 days) of drought exposure with subsequent restoration of normal irrigation, the plants of both varieties were characterized by the end of vegetation with a decline (by 16% for E70 and 20% for SY) above ground lengths in comparison with control non-stressed plants (Figure 3).

Seed priming with *B. subtilis* 104 and 26D in different levels decreased (by 40–50%) the negative impact of HDS on growth and contributed to faster recovery after the resumption of normal irrigation (Figure 3). Particularly, priming with bacteria (strains 104, 26D) was found to improve the intensity of growth processes (roots (by 128% and 130%) and shoots (by 137% and 135%) length, and their fresh and dry weight (by about 1.3–1.5 times)) of E70 and SY wheat varieties, respectively, under the combined effects of HDS in comparison with non-primed plants (Figure 3, Figure 4 and Figure 5). All groups of plants primed with 104 and 26D and exposed to HDS by the end of vegetation had higher above ground lengths, which even exceeded the length of control non-primed and non-stressed plants. Moreover, it was observed that E70 variety responded better to priming with strain 104, while SY to priming with strain 26D. In addition, 104 showed a more pronounced increase in the root length of E70 in comparison with 26D under HDS (Figure 4A,C). Additionally, it was established that in 26D-primed plants, the soil was firmly attached to the roots (Figure 5A). The results indicate the involvement of tested bacteria in modification of root architecture (by elongation of length) and regulation of the processes of more close interaction formation with the soil, playing a major role in supplying the plants with mineral nutrition.

Only the herbicide treatment did not have an inhibiting effect on growth and did not cause visible changes in plant phenotype, except for any yellowing of old leaves (data not presented). At the same time, the growth (above ground part) of plants primed with bacteria (strains 104, 26D) and sprayed with herbicide under normal conditions exceeded the growth parameters of plants with only herbicide and without bacterial treatment.

Representative photographs of visual appearance of bacterial-primed and non-primed (control) wheat plants of both varieties sprayed with herbicide and exposed to soil drought for 4 days and 7 days with subsequent restoration of normal irrigation are presented in Figure 3. Drought caused typical phenotypic changes in plants that were non-primed with bacteria and sprayed with herbicide and depended on the duration of stress exposure (Figure 3). An apparent plant wilting and stunting of growth was observed after 7 days of drought exposure for both wheat varieties. The number and degree of dead and yellowish leaves were more pronounced under a combination of HDS than in the separate effects of drought alone (not presented). In 4 days after the resumption of normal irrigation of the plant in all groups (with and without herbicide), which were subjected to drought, the plants began to restore turgor and resume growth. Bacterial treatment accelerated plant recovery for both varieties. It was also observed that strain 104 had the most significant accelerated recovery of E70 variety, while strain 26D—SY variety.

### 2.4. Chlorophyll Content and Leaf Area

**Figure 6:**
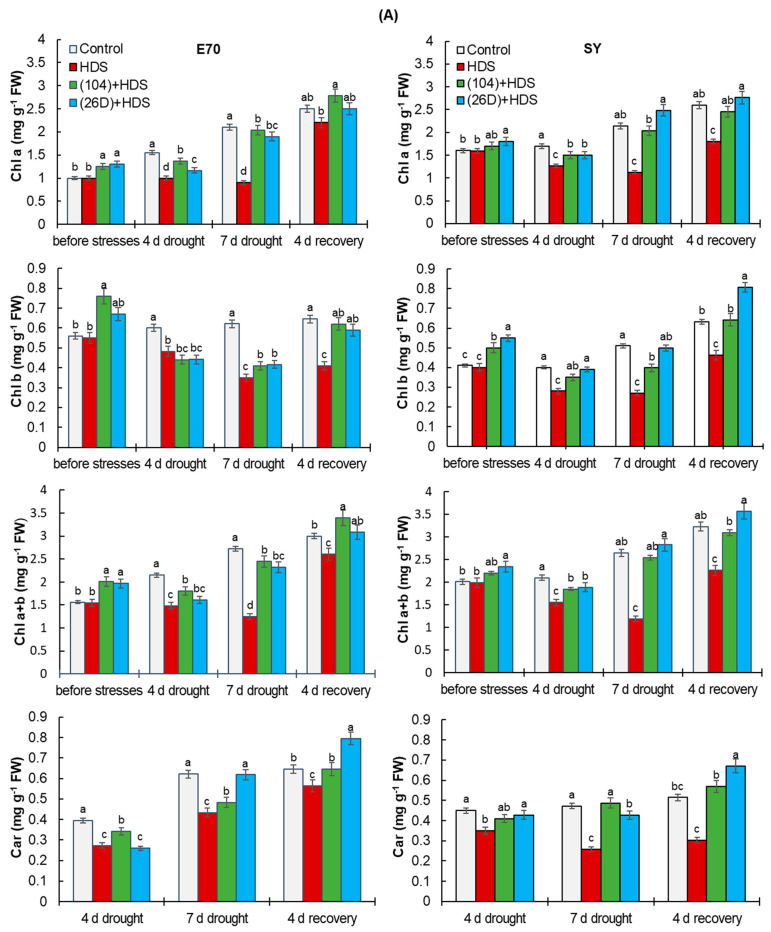

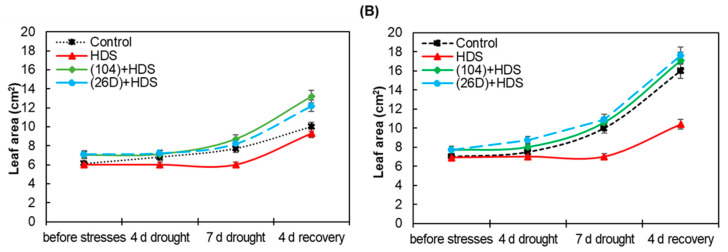
Influence of *B. subtilis* 104 and 26D on leaf photosynthetic pigments chlorophyll (Chl) a, Chl b (**A**) and leaf area (**B**) of wheat varieties E70 and SY under combined herbicide + drought stress (HDS), as well as in the post-stress period (4 days recovery after the resumption of normal watering). Error bars represent standard errors (±SE) of the means in triplicate. Growth parameters were measured using 25 plants from each replicate. Various letters show a significant difference between the averages of different groups at *p* < 0.05. HDS—herbicide + drought stress.

Chlorophyll (Chl) a and Chl b contents were increased in stressed wheat plants primed with strains 104 and 26D compared with non-primed plants. *B. subtilis*-inoculated and stressed plants showed higher values of total chlorophyll (Chl a + Chl b) content in comparison with uninoculated (control) stressed plants (Figure 6A). A significant decrease in total chlorophyll content (Chl a + Chl b) was observed in HDS-exposed plants in comparison with control ones. Application of *B. subtilis* strains (104, 26D) resulted in an increase in total chlorophyll content under HDS condition, respectively, compared with uninoculated (control) stressed plants (Figure 6A). Leaf area was changed similarly (Figure 6B).

### 2.5. Lipid Peroxidation (Malondialdehyde Content)

**Figure 7:**
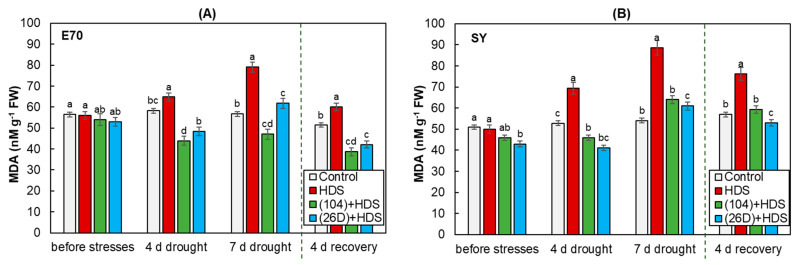
Influence of *B. subtilis* 104 and 26D on malondialdehyde (MDA) concentration in spring wheat varieties E70 (**A**) and SY (**B**) under combined herbicide + drought stress (HDS), as well as in the post-stress period (4 days of recovery). Error bars represent standard errors (±SE). Various letters show a significant difference between the averages of different groups at *p* < 0.05.

The results showed that HDS enhanced MDA generation in uninoculated wheat plants of both varieties. Exposure of herbicide-sprayed plants to drought over 7 days increased the content of MDA by about 1.5 and 2 times in plants of E70 and SY varieties, respectively (Figure 7). With that in SY plants, it was observed that the degree of damaging stress action after 4 and 7 days were expressed more strongly than for E70. Additionally, after restoring normal irrigation, the content of MDA in E70 plants reduced faster than in SY, demonstrating better recoverability of E70 in the post-stress period. Strains 104 and 26D significantly decreased HDS-induced MDA generation during 4 and 7 days of stress exposure in both varieties, demonstrating the most pronounced protective effect for E70. Compared with uninoculated plants, the reduction in MDA content after 4 days of normal irrigation in *B. subtilis*-inoculated stressed plants of both varieties were significant and reached control values for SY, and were even lower than control values for E70.

Under normal growth conditions (before exposure to stresses), both wheat varieties primed with strains 104 and 26D characterized with a slight (not significant) decrease in MDA concentration in comparison with uninoculated control wheat plants.

### 2.6. Proline Concentration

**Figure 8:**
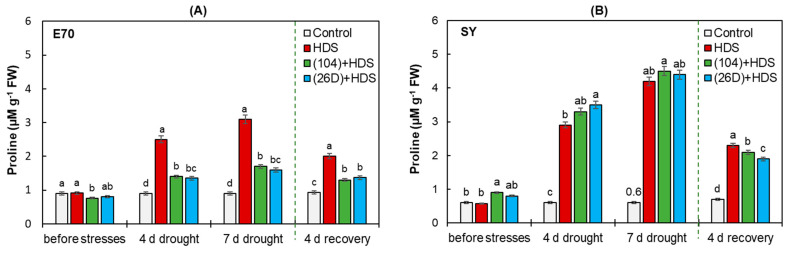
Effect of *B. subtilis* 104 and 26D on proline content in two wheat varieties E70 (**A**) and SY (**B**) under combined herbicide + drought stress (HDS) and after 4 days of normal irrigation (recovery). Error bars represent standard errors (±SE). Various letters show a significant difference between the averages of different groups at *p* < 0.05.

Exposure to HDS for 4 and 7 days resulted in a gradual increase (up to 2–4 times) in proline concentration in wheat plants of both varieties with the most pronounced effect for SY after 7 days of stress (Figure 8). Priming with 104 and 26D reduced (by 1.5–1.7 times) the level of stress-induced proline accumulation in E70 plants; while for SY variety, it led to an additional proline increase (up to 1.1 times) during 7 days of stress exposure. It has been established that after restoring normal irrigation, the decrease in stress-induced proline were more significant in bacterial-primed plants of both varieties, but the most pronounced effect was observed in E70. This generally indicates an increase in the adaptive capacity of plants and a more rapid recovery during the post-stress period upon application of seed priming with strains 104 and 26D.

### 2.7. Grain Yield Parameters

Exposure to HDS reduced the length of the spikes, number of grains per spike, and a mass of 1000 grains and grain yield per plant compared to control plants was observed (Table 2). Strains 104 and 26D differently influenced these parameters both under normal and HDS conditions. For plants of SY, when using 26D, there was a more noticeable increase in the length of the spike, the mass of 1000 grains, and grain yield per plant compared with 104 application both under normal and HDS; while for plants of E70 variety, the best indicators were noted when using strain 104. It should be noted that in plants exposed to HDS, the presence of deformed and shortened ears, as well as unfulfilled grains, was observed. While all bacteria-inoculated plants were characterized by the formation of normal spikes and grains. In addition, it was observed that the formation of spikes and maturation of seeds occurred earlier in inoculated plants of both varieties (on average, 4–5 days earlier than in uninoculated control).

## 3. Discussion

Plants and bacteria have established synergy interactions to mitigate harmful stresses [33,48]. The use of PGPMs as a seed biopriming agent is a promising eco-friendly tool for crop improvement. Moreover, the specificity of microbial strains’ action on different wheat varieties under drought was shown. For example, *B. safensis* W10 and *Ochrobactrum pseudogregnonense* IP8 treatment had a growth-stimulating and protective effect on six different varieties of drought-affected wheat, reflected in increased root mass, shoots, and grain yield [49]. Similar data were obtained from other bacterial strains (*Pantoea alhagi*, *Risobium leguminosarum*, *R. phaseoli*, *Mesorhizobium ciceri*, *Azospirillum brasilense*, *A. lipoferum*) on wheat, affected by drought at different stages of development, including the flowering period [50,51,52,53]. With regard to the combination of drought and herbicide stress, such information in the literature is limited. Recently, 11 bacterial isolates with characteristics of anti-stress agent properties were selected to mitigate the effects of herbicide stress and drought stress (water scarcity) on crops [54]. The isolated microorganisms were resistant to the action of a number of herbicides based on 2.4-D and sulfonyl urea, synthesized phytohormones, mobilized P compounds, had a potential for N fixation, and the isolates DA1.2 and H5%1 also had significant antifungal activity. It was demonstrated that the bacterial strain *P. avellanae* 6CH2 is stable and can grow at a high rate in the medium containing synthetic auxin-based herbicides (Octapon, 10 mL^−1^ L; Chistalan, 5 mL^−1^ L) and sulfonylureas (Nanomet, 0.05 g^−1^ L) [54]. Moreover, inoculation with *P. vellanae* 6CH2 exert an anti-stress effect on wheat plants which are sprayed with herbicides based on synthetic auxins 2.4-D, dicamba (Octapon, Chistalan), and metsulfuron-methyl (Nanomet) [45]. We established that both strains tested in our study are herbicide (Sekator^®^ Turbo) and drought (PEG-6000) tolerant (Figure 1) as well as capable to improve (in different degrees) wheat seed germination and early hydroponic seedling growth of both DT and DS varieties under different degrees of herbicide and drought stresses (Table 1, Figure 2). Regarding the influence of PGPMs on wheat plants differing in drought susceptibility under combined HDS during vegetation, underlying mechanisms were absent by the time we started our work. Our further results of pot experiments demonstrate that HDS-stressed plants of both varieties (DT and DS) primed with *B. subtilis* 104 and 26D maintain higher growth parameters in comparison with HDS-stressed plants without bacterial priming (Figure 3, Figure 4 and Figure 5). After the resumption of normal irrigation of plants in all variants (with and without herbicide), which were subjected to drought, the plants began to restore turgor and resume growth. Bacterial priming accelerated plant recovery. It should be noted that only the herbicide Sekator^®^ Turbo treatment under normal conditions did not have an inhibitory effect on plant growth and did not cause visible changes in plant phenotype, except for any yellowing of older leaves. Obviously, this is due to the fact that the herbicide Sekator^®^ Turbo contains an antidote mefenpirdiethyl, designed to mitigate the phytotoxic effect of the herbicide substances iodomulfuronmethyl sodium and amydromilfuron plants. It has been reported that mefenpirdiethyl enhances the activity of degradation enzymes and accelerates the decay of active agents of herbicide in plant tissues. The ability of wheat to tolerate the effects of some selective herbicides was also reported earlier [7], which was reflected in the absence of their inhibitory effect on plant growth, which is consistent with our findings.

One of the main processes of primary plant metabolism that is directly related to plant productivity is photosynthesis [4,8]. We observed increases in photosynthetic pigments chlorophyll a, b contents, and leaf area in bacterial-primed plants of both varieties under HDS conditions (Figure 6). Our results demonstrate the effectiveness of *B. subtilis* 104 and 26D to protect the photosynthetic pigments of wheat plants from the damaging effects of HDS, and indicate that one of the mechanisms of protective action of *B. subtilis* on wheat plants in stressful conditions caused by herbicide pressing and drought is their ability to positively regulate the photosynthetic apparatus of plants. Previous studies have also indicated that PGPMs’ application boosts the photosynthetic pigments in herbicide- stressed plants [38,45]. Particularly, *P. vellanae* 6CH2 increased the total chlorophyll (by 1.19–1.26 times) and decreased stress-caused proline in wheat plants against the background of herbicide stress [45]. This could be connected with higher accessibility and uptake of nutrients from the rhizosphere, which helps maintain plant growth under stress conditions [26,39]. Bourahla et al. [38] established the ability of *Pseudomonas putida* to improve some physio-biochemical parameters (i.e., chlorophyll, carotenoids, MDA, enzymatic activity) and reduce herbicide-caused oxidative stress in hydroponically grown wheat seedlings. In other studies, herbicide-tolerant and P-solubilizing *Burkholderia cepacian* PSBB1 reduced glyphosate toxicity, increased size, dry matter, nodule formation ability, nutrient content in chickpea seeds, and reduced the levels of proline and MDA [39]. The positive influence of PGPMs on photosynthesis of wheat plants under individual drought stress were also frequently reported [20,25].

Environmental stresses resulted in overproduction of reactive oxygen species (ROS), which at high levels can cause oxidative damage, impair membrane lipid functions, inactivate enzymes, and impede metabolic activities [55]. As a result, oxidative stress in cells accumulate a large quantity of denatured proteins and lipid peroxidation (LPO) products, which can be not only primary mediators of stress exposure, but also inductors of appropriate protective mechanisms in plant cells. Thus, the development of plant-protective reactions can be judged by the degree of accumulation of the final product of LPO—MDA [55]. In many studies in stressed plants, the contents of MDA notably declined thanks to PGPMs treatment [35,37,56,57]. The findings indicate that PGPMs alter the rate of metabolism by activating a strong ROS scavenging system in stressed plants. These may accelerate the restoration of membrane stability and protect against photodamage during re-watering in bacterial-treated plants [57]. In the present study, we observed that MDA levels were clearly higher in stressed plants of both varieties, which agrees with a previous report [58]. HDS exposure has been found to lead to significant increases in MDA levels in both wheat varieties, indicating intensive oxidative stress development and reduced plant resistance to stress (Figure 7). With that, for plants of SY variety, the degree of damaging stress action after 7 days of HDS exposure was more pronounced than for E70. Bacterial priming significantly reduced the high MDA levels in stressed plants. Therefore, the bacterial strains might suppress ROS production and prevent cell membrane damages under stress [58].

An important biochemical marker of plant tolerance formation can be the accumulation of proline—a multifunctional stress metabolite of plants, acting as an antioxidant, osmolyte, and low molecular chaperone, involved in maintaining the native structure of enzymes [55]. Previous reviews have reported that amino acid proline content increases in plants under stress [59,60]. Proline has been reported to play an important role in plant growth and life cycle by regulating cyclin genes, general protein synthesis [61], as well as in coordinating lignin biosynthesis [62]. The application of PGPMs rescued proline content in many cases in stressed seedlings during the recovery period. Proline also may play a role as a reservoir of organic N that can be consumed during the recovery period to help plants withstand environmental challenges [59,60]. There are contradictory data in the literature on the effect of PGPMs on osmolytes of different plants and under different stress factors. For example, inoculation with *B. cereus* BST YS1_42 increased proline in plants under normal condition, decreased under stress, and had a protective effect on growth [56]. Several reports have shown that the inoculation of plants with PGPMs including *B. subtilis* elevated proline content in plants under drought, thus increasing growth, biomass accumulation, and leaf water potential during stress [25,37,63]. The ambiguous nature of the relationship between the content of proline and the stress resistance of plants may be due to the different strength of stress effects in different experiments and the complex interaction of proline with other stress-protective systems, in particular, with the enzymatic antioxidant. In our experiments, a different degree of proline involvement in the formation of tolerance of studied wheat varieties under the influence of HDS and bacterial treatments was revealed. Particularly, priming with strains 104 and 26D reduced the level of stress-induced proline accumulation in plants of E70 and led to an additional increase in plants of SY. Additional proline accumulation in SY variety is suggested to contribute to the stabilization of the antioxidant system and ROS neutralization under HDS. The different responses of the two studied wheat varieties to priming with 104 and 26D indicated that proline is the most likely involved in the implementation of the genetically programmed tolerance of SY variety to the possible violation of the water regime and contributes less to the development strategy of E70 variety. The restoration of normal irrigation reduced stress-induced proline accumulation (down to the levels of control unstressed groups); this indicates an increase in the adaptive capacity of plants and a faster recovery during the post-stress period. Overall, our results suggest that osmotic regulation by strains 104 and 26D is an important cellular response helping plants to tolerate damages caused by herbicide and soil drought.

Grain yield is a cumulative indicator of physio-biochemical processes for the entire period of plant vegetation. It was reported about the influences of PGPM-based biopreparations on wheat grain yield/quality under different mineral nutrition backgrounds [64,65,66]. However, there are limited information in the literature about the influence of PGPMs on wheat grain yield under combined drought and herbicide stress. So, the influence of *Pseudomonas protegens* (DA1.2)-based biopreparation Agrobiolog on yield and quality indicators of wheat grain under conditions of moderate drought against the background of the action of herbicides of different chemical composition is shown [43]. *P. protegens* DA1.2 application increased wheat yield by 17–36% and increased protein and gluten grade from 3rd to 2nd grade (in herbicide-treated plants) and to 1st grade (in plants without herbicide treatment) [43]. However, the information about the effect of PGPMs in these conditions on different wheat cultivars, especially contrasting in drought sensitivity, is absent. To the best of our knowledge, this is the first study which showed that seed priming with *B. subtilis* 104 and 26D improved the length of the spikes, number of grains per spike, mass of 1000 grains, and grain yield compared to non-primed plants under HDS conditions (Table 2). Moreover, the findings suggest that for plants of SY, when using strain 26D, there was a more noticeable increase in the length of the spike, the mass of 1000 grains, and grain yield compared with strain 104 application both under normal and HDS; while for plants of E70 variety, the best indicators were noted when using strain 104. In addition, it was observed that the formation of ears and maturation of seeds occurred earlier (about 4–5 days earlier than in non-primed control plants of both varieties). Thus, revealing the mechanisms of PGPM plays a key role in determining the most effective ways to use them to improve grain yield/quality and minimize the damaging effects of stresses, including herbicide and drought on wheat plants. Further research should be focused on understanding the mechanisms that determine the strain and variety-specificity of endophytic symbiosis and the role of bacteria in the modulation of the physiological status of inoculated wheat plants under such stressful conditions such as herbicide and drought stresses.

## 4. Materials and Methods

### 4.1. Plant Materials and Bacterial Strains

The experiments were conducted in *Triticum aestivum* L. (spring wheat) varieties Ekada70 (E70) and Salavat Yulaev (SY) previously identified as tolerant and susceptible to drought, respectively [37]. The seeds were supplied by the Chishmy Breeding Station, Ufa Federal Research Center, Russian Academy of Sciences (UFRC RAS) (Chishmy, Russia). The endophytic bacterial strain *B. subtilis* 104 was previously isolated from the arable soils of the Republic of Bashkortostan (Russia) at the Bashkir Research Institute of Agriculture UFRC RAS (Ufa, Russia) and characterized in detail [32,35]. Based on the results of the sequencing analysis of the variable regions of genes encoding 16S rRNA and PCR analysis using species-specific primers (secYsubF TTATATCACGGCTTCGAT, secYsubR CGGTAGTTTCGTTTCACCA), the strain 104 was identified as *B. subtilis* [32] and deposited in the National Bio-Resource Center of the All-Russian Collection of Industrial Microorganisms (VKPM) (registration number B-12988). The endophytic bacterial strain *B. subtilis* 26D (registration number 016-02-2491-1) (etalon strain) is the basis of the commercial biological product Phytosporin-M (BashInkom S&IE, Ltd., Ufa, Russia) and was kindly provided by the Microbiological Laboratory of BashInkom S&IE, Ltd. (Ufa, Russia).

### 4.2. Preparation of Bacterial Inoculum Suspensions and Seed Priming

*B. subtilis* cells were cultivated in Luria–Bertani (LB) medium (at 37 °C, 180 rpm) for 24 h until the cell concentration reached 10^9^ colony-forming units (CFU) mL^−1^. Then the bacterial suspensions of strain 104 and 26D were diluted down to 10^5^ CFU mL^−1^ (selected previously as optimal for promoting growth and protecting plants from stresses) [32,35] and 10^8^ CFU mL^−1^ (as recommended by manufacturer) using sterile water. The cells’ concentration was determined at 600 nm (SmartSpecTM Plus spectrophotometer, Bio-Rad, Hercules, CA, USA).

The seeds were sterilized in 97% ethyl alcohol for 60 s, washed five times with tap water, and then immersed into suspensions of *B. subtilis* 10−4 (10^5^ CFU mL^−1^), *B. subtilis* 26D (10^8^ CFU mL^−1^) or water (control) for 1 h.

### 4.3. Herbicide

The commercially sold herbicide Sekator^®^ Turbo was used in this work. The Sekator^®^ Turbo herbicide (Bayer AG, Frankfurt, Germany) contains active ingredients iodosulfuron methyl sodium (25 g L^−1^), amidosulfuron (100 g L^−1^), and mefenpyrdiethyl (antidote) (250 g L^−1^). The doses used in bacteria and early hydroponic seedling growth tests were calculated taking into account the doses recommended by manufacturers to use in wheat fields (50–100 mL ha^−1^ with working solution consumption 200 L ha^−1^) and were 0.25 mL L^−1^ (Hb50), 0.375 mL L^−1^ (Hb75), and 0.5 mL L^−1^ (Hb100) which corresponds to 50, 75, and 100 mL ha^−1^, respectively. The dose used in pot experiments was 100 mL ha^−1^ (recalculated as 0.5 mL L^−1^, working solution sprayed as 200 L ha^−1^).

### 4.4. Herbicide Stress Tolerance of Bacterial Strains

The herbicide tolerance of bacterial strains was assessed in triplicate by observing their growth on liquid LB medium supplemented with different Sekator^®^ Turbo herbicide concentrations (0.25 mL L^−1^ (Hb50), 0.375 mL L^−1^ (Hb75), and 0.5 mL L^−1^ (Hb100)). The bacterial growth in liquid LB media was monitored by measuring the optical density at 600 nm using SmartSpecTM Plus spectrophotometer (Bio-Rad, Hercules, CA, USA). The significant growth of bacterial strains in the presence of herbicide during 24 h at 37 °C (180 rpm) was considered as herbicide tolerant.

### 4.5. Drought Stress Tolerance of Bacterial Strains

The drought tolerance of bacterial strains was examined on liquid LB medium supplemented with varied polyethylene glycol (PEG-6000) concentrations (0, 4, 8, and 12% resulting in osmotic potentials of 0, 0.10, 0.20, and 0.45 MPa, respectively). The bacteria were incubated at 37 °C (180 rpm) for 24 h. The bacterial growth in liquid LB media was monitored by measuring the optical density at 600 nm (SmartSpecTM Plus, Bio-Rad, Hercules, CA, USA).

### 4.6. In Vitro Seed Germination and Early Hydroponic Seedling Growth Assays

To assess the herbicide and drought tolerance of seeds during germination and early seedlings’ growth, we measured the ability of seeds to germinate and grow in solutions of Sekator^®^ Turbo herbicide (0.25 mL L^−1^ (Hb50), 0.375 mL L^−1^ (Hb75), and 0.5 mL L^−1^ (Hb100)), and PEG-6000 (0, 4, 8, and 12%). The bacterial-primed and non-primed seeds were sown in Petri dishes with 5 mL of herbicide and/or PEG-6000 solutions (tests) and water (control) (15 seeds per dish, three replicates). The seeds were grown for 3 and 7 days in the dark at 22 °C after which the number of germinated seeds was counted (on 3 and 7 days) and growth parameters (length, fresh biomass) (on 7 days) was measured. The percentage of germination was determined by the number of seeds giving a rootlet of the smallest length [67].

### 4.7. Pot Experiment Design, Treatments, and Growth Conditions

Bacterial-primed (test) and non-primed (control) seeds were grown on filter paper with tap water for three days under a long-day photoperiod (16 h light/8 h dark, 22–24 °C). Thereafter, 3-day-old seedlings were transferred to pots (30 × 30 × 30 cm) with soil (optimal NPK ratio, pH 6.5, humidity 65%) (LLC Veltorf, Velikie Luki, Russia). Distance between plants in rows were 3 cm; distance between rows were 7–8 cm. On the bottom of each pot was pre-laid agrotechnical clay (size 10–20 mm) (Florizel, Terra Master Ltd., Krasnoyarsk, Russia) and then each was filled with soil. After planting, wheat plants were grown under controlled conditions (16 h light/8 h dark, 22–24 °C, 15,000 lux lighting, RH 60%). During the first 17 days, all groups of plants were watered 2 times a week. Further, 17-day-old plants (phase 2–3 leaf) were divided into 2 groups: (1) grown normally and under normal irrigation; (2) subjected to herbicide pressing, for the simulation of which, part of the plant pots were sprayed once with a solution of the highly selective herbicide Sekator^®^ Turbo (Bayer AG, Frankfurt, Germany) with a flow rate of 75 mL ha^−1^, according to the manufacturer’s recommendations (https://www.cropscience.bayer.ru/product/sekator-turbo (accessed on 1 June 2021)). Soil drought was simulated in 4 days after spraying of the 17-day-old wheat plants with herbicide by stopping irrigation of the plants for 7 days (until the water deficit was reached, 60% lower than the normal irrigated control groups) with subsequent resumption of normal irrigation. Each variant was carried out in three replicates with 25 plants per replicate.

Morphological and physiological assessment of the state of the plants was carried out in dynamics at 4 points: (1) before exposure to stresses (16-day-old plants, 3–4 leaf phase); (2) after 4 days of drought exposure; (3) after 7 days of drought exposure; (4) 4 days after the resumption of normal irrigation (recovery) [7,8].

### 4.8. Determination of Photosynthetic Pigments and Leaf Area

Photosynthetic pigments chlorophyll (Chl) a and Chl b were assayed as described [68]. Briefly, ground plant leaves (0.05 g) were extracted in 90% ethanol (10 mL) with the addition of CaCO_3_. Pigment absorption was then measured at 663 (Chl a) and 646 (Chl b) (SmartSpecTM Plus, Bio–Rad, Hercules, CA, USA).

The leaf area was estimated by using scanner image analysis. Plant leaves were placed in an optical scanner HP laser MFP 135 w (HP Inc., Paolo Alto, CA, USA), covered with a sheet of thick white paper, and scanned with a resolution 200 dpi in a black and white halftone image mode. Leaf area obtained in pixels was converted to cm^2^ (http://csaa.ru/opredelenie-ploshhadi-listev-metodom-skanirovanija/ (accessed on 12 April 2022)). In each variant, three leaves in three replicates were estimated.

### 4.9. Lipid Peroxidation (LPO) Assay

LPO degree was assessed by malondialdehyde (MDA) content [69]. The ground samples (0.5 g) were homogenized in dH_2_O (3 mL), with the subsequent addition of 20% trichloroacetic acid (3 mL), followed by centrifugation at 10,000× *g* for 10 min. The supernatant (2 mL) was mixed with 0.5% thiobarbituric acid (2 mL), and the mixture was heated at 100 °C for 30 min, then rapidly cooled. The optic density of the obtained solution was measured at 532 nm and 600 nm (SmartSpecTM Plus, Bio-Rad, St. Louis, MO, USA).

### 4.10. Determination of Proline Content

The content of proline was evaluated using ninhydrin reagent [70]. Plant samples (0.5 g) were ground with 5 mL of boiling distilled water, incubated at 100 °C for 30 min and cooled. Then 1 mL of the extract was mixed with 1 mL of ninhydrin reagent and 1 mL of acetic acid, incubated at 100 °C for 1 h and cooled. Optical density of solutions was measured at 522 nm (SmartSpecTM Plus, Bio-Rad, St. Louis, MO, USA).

### 4.11. Evaluation of Grain Yield

Grain yield parameters were evaluated in all plants of two wheat varieties studied in both non-stressed (control) and stressed conditions, and primed with bacterial strains 104 and 26D. The length of spikes, number of grains per spike, 1000 grain weight, and grain yield were assessed in the first spike of each plant [71].

### 4.12. Statistical Analysis

All physiological and biochemical experiments were carried out in three biological and three analytical replicates. The results represented the average values of the three replicates as the mean ± standard error (SE). Statistically significant differences between the mean values were estimated using an analysis of variance (ANOVA), followed by the Tukey test (*p* < 0.05).

## 5. Conclusions

Overall, the results indicate the effectiveness of seed priming with endophytic strains *B. subtilis* 104 and 26D to improve growth, increase photosynthetic pigment content and leaf area, and to reduce oxidative and osmotic cell damages in wheat plants (differing in susceptibility to dehydration) from the damaging effects of HDS, as well as faster recovery of primed plants in the post-stress period, which eventually manifests itself in better grain yield formation. The existence of varietal and strain-specific efficacy of endophytic *B. subtilis* on growth, development of protective reactions, and grain yield formation in two wheat varieties (differing in drought susceptibility) under both normal and combined HDS was observed. Strain 104 had a pronounced growth-stimulating and protective effect on the growth and metabolism of E70 variety plants, while strain 26D—for SY variety plants.

## Figures and Tables

**Table 1:** Effect of various concentrations of Sekator^®^ Turbo-induced herbicide stress and PEG-induced drought stress on seed germination and early seedling growth in wheat (*Triticum aestivum* L.) pre-treated before sowing with *Bacillus subtilis* (strains 104 and 26D). In the table, standard errors (±SE) of the means in triplicate (15 seeds/seedlings per replicate) are represented. Various letters show a significant difference between the averages of different groups at *p* < 0.05.

Concentration of PEG (%)	3rd Day		7th Day		Shoot Length (cm)		Root Length (cm)	
	Germination (%)		Germination (%)					
	E70	SY	E70	SY	E70	SY	E70	SY
0 (control)	99 ± 1 ^a^	80 ± 3 ^a^	99 ± 1 ^a^	82 ± 2 ^a^	6.8 ± 0.4 ^a^	5.3 ± 0.3 ^a^	5.8 ± 0.2 ^a^	5.4 ± 0.1 ^a^
4	84 ± 2 ^d^	73 ± 2 ^c^	84 ± 2 ^d^	73 ± 2 ^c^	5.7 ± 0.5 ^c^	4.9 ± 0.2 ^c^	5.2 ± 0.1 ^c^	4.8 ± 0.2 ^d^
4 + (strain 104)	93 ± 2 ^b^	80 ± 1 ^a^	95 ± 1 ^b^	82 ± 1 ^ab^	6.2 ± 0.3 ^b^	5.0 ± 0.3 ^bc^	5.9 ± 0.3 ^a^	4.9 ± 0.3 ^c^
4 + (strain 26D)	86 ± 3 ^c^	76 ± 2 ^b^	88 ± 3 ^c^	78 ± 5 ^b^	6.1 ± 0.2 ^b^	5.1 ± 0.2 ^b^	5.6 ± 0.2 ^b^	5.2 ± 0.4 ^b^
8	72 ± 1 ^g^	67 ± 4 ^e^	73 ± 2 ^e^	67 ± 2 ^e^	4.9 ± 0.1 ^e^	4.0 ± 0.5 ^f^	4.3 ± 0.3 ^e^	3.7 ± 0.2 ^f^
8 + (strain 104)	87 ± 2 ^e^	70 ± 1 ^d^	88 ± 1 ^c^	70 ± 3 ^d^	5.3 ± 0.6 ^d^	4.5 ± 0.3 ^de^	5.1 ± 0.4 ^c^	4.2 ± 0.3 ^e^
8 + (strain 26D)	84 ± 2 ^f^	69 ± 1 ^d^	84 ± 2 ^d^	69 ± 4 ^d^	5.0 ± 0.7 ^de^	4.6 ± 0.2 ^d^	4.9 ± 0.1 ^d^	4.8 ± 0.4 ^d^
12	68 ± 3 ^k^	60 ± 2 ^g^	68 ± 1 ^g^	60 ± 1 ^j^	3.8 ± 0.5 ^j^	3.1 ± 0.4 ^j^	3.8 ± 0.2 ^g^	2.9 ± 0.3 ^j^
12 + (strain 104)	72 ± 1 ^g^	63 ± 3 ^f^	73 ± 3 ^e^	64 ± 2 ^f^	4.2 ± 0.6 ^f^	3.7 ± 0.1 ^g^	4.4 ± 0.5 ^e^	3.2 ± 0.2 ^g^
12 + (strain 26D)	70 ± 2 ^j^	62 ± 1 ^f^	71 ± 2 ^f^	62 ± 3 ^g^	4.0 ± 0.3 ^fg^	3.9 ± 0.1 ^f^	4.1 ± 0.3 ^f^	3.7 ± 0.2 ^f^
**Concentration** **of Sekator^®^ Turbo**	**3rd day**		**7th day**		**Shoot Length** **(cm)**		**Root Length** **(cm)**	
	**Germination** **(%)**		**Germination** **(%)**					
	**E70**	**SY**	**E70**	**SY**	**E70**	**SY**	**E70**	**SY**
Hb0 (control)	98 ± 2 ^a^	86 ± 4 ^a^	98 ± 1 ^a^	86 ± 3 ^a^	4.2 ± 0.2 ^a^	4.6 ± 0.1 ^a^	6.1 ± 0.2 ^a^	6.6 ± 0.3 ^a^
Hb50	53 ± 4 ^d^	27 ± 5 ^f^	55 ± 2 ^f^	25 ± 5 ^f^	1.7 ± 0.1 ^d^	0.5 ± 0.3 ^d^	0.9 ± 0.1 ^e^	0.1 ± 0.05 ^f^
Hb50 + (strain 104)	76 ± 3 ^b^	67 ± 2 ^b^	78 ± 1 ^b^	67 ± 2 ^b^	2.2 ± 0.3 ^b^	0.9 ± 0.2 ^bc^	1.5 ± 0.2 ^b^	0.8 ± 0.1 ^c^
Hb50 + (strain 26D)	67 ± 5 ^c^	53 ± 3 ^c^	68 ± 3 ^d^	54 ± 2 ^c^	1.9 ± 0.2 ^c^	1.0 ± 0.1 ^b^	1.2 ± 0.2 ^c^	1.3 ± 0.1 ^b^
Hb75	46 ± 1 ^f^	23 ± 4 ^g^	47 ± 4 ^g^	14 ± 1 ^k^	1.3 ± 0.1 ^f^	0.3 ± 0.1 ^e^	0.7 ± 02 ^f^	0.05 ± 0.01 ^g^
Hb75 + (strain 104)	73 ± 3 ^bc^	46 ± 2 ^d^	76 ± 2 ^c^	49 ± 1 ^d^	1.7 ± 0.1 ^d^	0.5 ± 0.1 ^d^	1.4 ± 0.1 ^bc^	0.3 ± 0.1 ^d^
Hb75 + (strain 26D)	60 ± 2 ^d^	33 ± 3 ^e^	63 ± 3 ^e^	37 ± 4 ^e^	1.5 ± 0.1 ^e^	0.4 ± 0.2 ^d^	1.1 ± 0.3 ^d^	0.2 ± 0.04 ^e^
Hb100	37 ± 2 ^j^	14 ± 1 ^k^	41 ± 2 ^k^	15 ± 2 ^k^	0.5 ± 0.2 ^k^	0.07 ± 0.03 ^g^	0.4 ± 0.1 ^g^	0.03 ± 0.01 ^j^
Hb100 + (strain 104)	48 ± 4 ^e^	19 ± 3 ^j^	48 ± 1 ^g^	19 ± 3 ^j^	1.0 ± 0.3 ^g^	0.2 ± 0.1 ^f^	1.0 ± 0.1 ^de^	0.1 ± 0.02 ^e^
Hb100 + (strain 26D)	43 ± 3 ^g^	23 ± 4 ^g^	43 ± 1 ^j^	24 ± 2 ^fg^	0.7 ± 0.1 ^h^	0.3 ± 0.1 ^e^	0.8 ± 0.2 ^ef^	0.2 ± 0.1 ^e^

**Table 2:** Effect of seed priming with *B. subtilis* 104 and 26D on the formation of yield elements of soft spring wheat under normal and HDS conditions. In the table standard errors (±SE) of the means in triplicate are represented. In each replicate, 25 plants were used. Various letters show a significant difference between the averages of different groups at *p* < 0.05.

Treatment	Plant Length (cm)	Spike Length (cm)	Grains Number per Spike	Mass of 1000 Grains (g)	Grain Yield (g per Plant)
E70 variety					
Control	45 ± 1.1 ^d^	4.8 ± 0.7 ^b^	8.1 ± 0.3 ^d^	23.7 ± 2.7 ^c^	0.19 ± 0.03 ^e^
104	51 ± 0.3 ^a^	5.0 ± 0.9 ^a^	11.7 ± 0.5 ^a^	25.6 ± 1.3 ^a^	0.30 ± 0.02 ^a^
26D	48 ± 0.6 ^bc^	4.9 ± 0.7 ^a^	10.8 ± 0.4 ^b^	24.4 ± 1.5 ^b^	0.26 ± 0.04 ^b^
HDS	38 ± 0.9 ^e^	4.1 * ± 0.4 ^d^	6.3 ± 0.3 ^e^	15.1 ± 2.6 ^e^	0.11 ± 0.06 ^f^
104 + HDS	52 ± 0.7 ^a^	4.7 ± 0.3 ^bc^	10.2 ± 0.6 ^b^	23.5 ± 1.9 ^c^	0.24 ± 0.04 ^c^
26D + HDS	50 ± 0.8 ^b^	4.5 ± 0.6 ^c^	9.9 ± 0.7 ^c^	22.3 ± 2.1 ^d^	0.22 ± 0.03 ^d^
SY variety					
Control	47 ± 0.8 ^d^	5.6 ± 1.3 ^c^	6.2 ± 1.7 ^d^	21.2 ± 1.6 ^c^	0.13 ± 0.02 ^c^
104	49 ± 0.9 ^c^	5.7 ± 0.9 ^b^	7.1 ± 1.1 ^b^	22.4 ± 0.6 ^b^	0.16 ± 0.01 ^b^
26D	54 ± 0.4 ^a^	5.9 ± 0.6 ^a^	8.4 ± 1.9 ^a^	23.6 ± 1.3 ^a^	0.20 ± 0.02 ^a^
HDS	37 ± 0.7 ^e^	3.4 * ± 1.3 ^f^	3.5 ± 0.7 ^f^	14.2 ± 1.5 ^f^	0.05 ± 0.03 ^e^
104 + HDS	46 ± 0.6 ^d^	4.5 ± 0.9 ^e^	5.6 ± 1.0 ^e^	17.2 ± 0.7 ^de^	0.10 ± 0.01 ^d^
26D + HDS	52 ± 0.2 ^b^	5.4 ± 1.2 ^d^	6.7 ± 1.5 ^c^	18.3 ± 0.3 ^d^	0.13 ± 0.01 ^c^

* Some spikes did not produce grain (were empty) and the presence of deformed spikes was observed.

## Data Availability

Not applicable.
